# The Success of Endodontic Treatments Performed by Dental Residents in Advanced Education in General Dentistry Program: A 10-Year Retrospective Study

**DOI:** 10.3390/dj13070306

**Published:** 2025-07-08

**Authors:** Nisreen Al Jallad, Eli Sun, Tongtong Wu, Shasha Cui, Amer Basmaji, Radhika Thakkar, Shahenda Aboelmagd, Neha Naik, Konstantina Tzouma, Jin Xiao, Hans Malmstrom

**Affiliations:** 1 Eastman Institute for Oral Health, University of Rochester Medical Center, Rochester, NY 14620, USA; nisreen_aljallad@urmc.rochester.edu (N.A.J.); shasha_cui@urmc.rochester.edu (S.C.); amer_basmaji@urmc.rochester.edu (A.B.); radhika_thakkar@urmc.rochester.edu (R.T.); shahenda_aboelmagd@urmc.rochester.edu (S.A.); neha_naik@urmc.rochester.edu (N.N.); konstantina_tzouma@urmc.rochester.edu (K.T.); jin_xiao@urmc.rochester.edu (J.X.); 2 Department of Biostatistics and Computational Biology, University of Rochester Medical Center, Rochester, NY 14642, USA; eli_sun@urmc.rochester.edu (E.S.); tongtong_wu@urmc.rochester.edu (T.W.)

**Keywords:** endodontic treatment, root canal therapy, postgraduate residency, dental education, treatment outcomes, patient satisfaction

## Abstract

**Objectives:** This study aimed to evaluate the success rates of NSRCT performed by AEGD residents and to identify predictive factors associated with clinical outcomes and patient satisfaction. **Methods:** A retrospective chart review was conducted on cases treated between 2012 and 2021. Eligible cases included fully developed permanent teeth that underwent NSRCT and received a final restoration by general dentistry residents, with a minimum of 12 months of follow-up. Data collected included demographic information, medical history, clinical symptoms, radiographic findings, instrumentation, type and timing of final restorations, and patient satisfaction scores. Treatment success was defined as the absence of symptoms and either the resolution or stability of periapical radiolucency. Patient satisfaction and pain levels were also analyzed. **Results:** AEGD residents achieved radiographic and clinical RCT success rates of 93.3% and 91.5%, respectively. Multivariate logistic regression showed that the presence of an intact restoration was significantly associated with increased odds of tooth retention (odds ratio [OR] = 3.4, *p* < 0.001), while post placement in a straight root was also a significant predictor of survival (OR = 4.2, *p* = 0.02). Conversely, pre-existing radiolucency (OR = 0.37, *p* = 0.018) and the use of a metal post (OR = 0.23, *p* = 0.012) were significantly associated with lower odds of tooth retention. Worse periodontal health was significantly associated with increased odds of pain on percussion, with a 74.19% increase in odds per worsening category (OR = 1.74, *p* = 0.002). Patient satisfaction was significantly higher when restorations remained intact, with esthetic satisfaction increasing by a factor of 3.08 (OR = 3.08, *p* < 0.001) and functional satisfaction increasing by a factor of 3.9 (OR = 3.9, *p* < 0.001). **Conclusions:** Endodontic treatments performed by AEGD residents demonstrated high success rates and favorable patient-reported outcomes. Restoration integrity, periodontal health, and post and final restoration selection play critical roles in treatment success and patient satisfaction.

## 1. Introduction

Preservation of the natural dentition remains an important outcome in securing oral health. Endodontic treatments have been shown to successfully retain compromised teeth that were fractured, carious, or traumatized for many decades [1]. According to the quality assurance guidelines of the American Association of Endodontics (AAE), the objective of root canal treatment (RCT) has been described as follows: (a) the prevention of adverse signs or symptoms, (b) the creation of radiographic appearance of a well obturated root canal system, (c) the promotion of healing and repair of peri-radicular tissues, (d) the prevention of further breakdown of peri-radicular tissues, and (e) the removal of contents from the root canals [2].

Many factors have been associated with the long-term success of endodontic therapy including but not limited to the absence of an apical lesion, the use of dental dams during endodontic treatment and core placement, the use of surgical operating microscopes, periodontal condition, structural integrity/restorability of the tooth, and effective post-endodontic restoration [3].

The evaluation of the success of RCT is based on the radiographic as well as clinical assessment [4]. Benenati and Khajotia (2002) reported a 91.05% combined percentage of success and acceptable rate in a radiographic recall evaluation of eight hundred ninety-four cases performed by students in Oklahoma Dental School, with follow-up periods ranging from 6 months to 7 years [5]. Alley et al. (2004) [6] conducted a chart review at three private general practices in Alabama, USA, to compare the success of endodontic treatment provided by specialists versus general dentists (GDs). Success was defined as the treated tooth being present 5 years after the date of treatment initiation. Calibrated dentists reviewed 350 charts, 195 teeth were treated by general dentists, achieving an 89.7% success rate, while 155 teeth were treated by endodontists, who achieved a 98.1% success rate. They concluded that endodontic treatment by specialists was significantly more successful [6]. The Toronto Study, conducted in a university graduate clinic setting, reported a long-term healing rate of 86% for endodontic treatment, based on the absence of apical periodontitis and symptoms over a 4–6 year follow-up period [7,8]. Burry et al. (2016) [3] compared the outcomes of initial nonsurgical root canal therapy for different tooth types provided by both endodontists and other providers. Significant differences in survival based on provider type were noted for molars at 5 years and for all tooth types at 10 years. The greatest difference discovered was 5% higher survival rate at 10 years for molars treated by endodontists [3].

A systematic review and meta-analysis of 42 studies showed that the weighted pooled success rates were estimated to be 92.6% (95% CI: 90.5–94.8%) under ‘loose criteria’, which was defined as a reduction in the size of the existing periapical lesion and 82.0% (95% CI: 79.3–84.8%) under ‘strict’ criteria, which was defined as the complete resolution of the periapical lesion [9]. Another interesting retrospective study used an insurance company database, aimed at assessing 110,766 nonsurgical root canal therapies completed by endodontists and general dentists, and found that the success rate was 94.44% over an average follow-up time of 3.5 years. The results from this study suggest that the clinical success of specialist practice was similar to the clinical success of general dentists, even with complex cases [10].

Research Gap: Evidence-based knowledge on the outcome of endodontic therapy is key to clinical decision making, particularly when endodontic treatment is weighed against tooth extraction and replacement [11]. However, most research focuses on endodontic treatments conducted by specialists, undergraduate students, or general dental practitioners. For instance, Touboul et al. (2014) assessed outcomes of treatments performed by postgraduate endodontic students, not general dentistry residents [12].

The literature lacks studies evaluating the outcomes of endodontic therapy performed by residents in postgraduate general dentistry residency programs. Investigating the clinical success of root canal therapy within such programs, particularly those with structured endodontic training and long-term follow-up, would provide valuable insights into treatment effectiveness and inform potential improvements in curriculum design. Addressing this gap, our study offers novel data on endodontic treatment outcomes achieved by residents in a postgraduate general dentistry setting.

Primary Aim: This study aimed to assess the success rate of endodontic treatments performed by residents under the supervision of faculty in the one and two-year Advanced Education in General Dentistry (AEGD) program at Eastman Institute for Oral Health (EIOH), University of Rochester, Rochester, NY. Success was considered the treated tooth retaining function and being asymptomatic in the oral cavity for at least 12 months following treatment completion and final restoration. Given that our study is conducted in a supervised academic setting and based on strong evidence from previous studies, reported success rates for nonsurgical root canal treatments ranged from 86% to 95% [8,9,13]. We hypothesize that the success rate of endodontic treatment performed by the AEGD residents in EIOH is equal to or higher than 92%.

The secondary aims of this study were as follows: (a) to assess patient satisfaction with endodontic treatment and restorations; (b) to identify potential factors influencing the outcomes of endodontic treatment; (c) to evaluate the success of root canal treatment in relation to variables such as tooth distribution, initial radiographic appearance, periapical status, pain, and type of restoration; (d) and to examine the association between root canal treatment success and patient medical history, including factors such as diabetes, smoking status, hygiene status, age, gender, and race/ethnicity.

## 2. Methods

### 2.1. Study Design

This retrospective chart review was conducted at the Eastman Institute for Oral Health (EIOH), University of Rochester. Research Subjects Review Board RSRB office of the University of Rochester determined on 13 March 2023 that this retrospective chart review protocol meets federal and University criteria for exemption (RSRB; STUDY0000809). Data were collected from electronic dental records (axiUm) for patients who received non-surgical root canal treatment (NSRCT) and subsequent final restoration by residents in the Advanced Education in General Dentistry (AEGD) program between 1 April 2012 and 31 December 2021.

### 2.2. Inclusion and Exclusion Criteria

Patients were eligible if they

1. Had permanent, fully developed teeth.
2. Underwent initial NSRCT by AEGD residents at EIOH within the specified date range.
3. Had complete radiographic documentation: pre-treatment periapical (PA) X-ray, PA X-ray at the completion of RCT, and a bitewing (if restored with a crown).
4. Had a documented recall examination (EXAM05) in axiUm.
5. Completed a minimum of 12 months of follow-up from the date of the final restoration to the documented recall visit.

Patients were excluded if

1. They had immature teeth at the time of treatment.
2. Their follow-up duration was less than 12 months.
3. Required radiographic documentation was incomplete or missing.

### 2.3. Resident Calibration and Data Quality

All recall exams (EXAM05) were performed by calibrated AEGD residents using a standardized electronic template. Calibration was ensured by comparing each resident’s assessments with a gold standard established by this study’s principal investigator (PI). The PI evaluated three sample patients, and each resident independently examined the same patients on three separate occasions spaced two weeks apart. Inter-examiner reliability was calculated to ensure consistency.

### 2.4. Data Collection and Variables

Demographic data included age, gender, and race/ethnicity. Medical history variables included ASA classification, smoking status, and systemic conditions (e.g., diabetes, asthma). Oral health status, including periodontal condition and hygiene, was also extracted.

Treatment-related variables included the following:

- Technique used: Hand filing, rotary instruments, or both.
- Tooth classification: Anterior, premolar, or molar; maxillary or mandibular.
- Radiographic data: Quality of obturation, presence of pre- and post-operative radiolucency.
- Pain scores: Using the Visual Analog Scale (VAS).
- Type of final restoration: Amalgam, composite, or crown.
- Timing of final restoration: Number of days between RCT completion and restoration placement.
- Patient-reported satisfaction: Esthetics, function, occlusion, overall experience (0–10 Likert scale).

### 2.5. Outcome Assessment

Treatment success was defined as follows:

- Clinical: Absence of pain, swelling, sinus tract, and sensitivity to percussion or palpation.
- Radiographic: Absence or reduction in periapical radiolucency.
- Functional: Retention of the treated tooth without complications for ≥12 months after restoration.

### 2.6. Criteria for Evaluation

The periapical index (PAI) scoring system developed by Orstavik and the team was used to assess the outcome of endodontic treatment [14]. Benjamin Baker stated in his systematic review that the use of the periapical index PAI scoring system developed by Orstavic seems to be the accepted valid tool for evaluating treatment outcome [15]. The PAI scoring system assigns scores based on radiographic assessment as follows: A score of 1.0 indicates that periapical destruction of bone is almost definitely not present, while a score of 2.0 suggests that it is almost probably not present. A score of 3.0 reflects uncertainty regarding the periapical destruction of bone based on the radiographic evaluation. A score of 4.0 indicates that periapical destruction of bone is probably present, and a score of 5.0 confirms that it is almost definitely present. Scores of 1.0 and 2.0 indicate a non-diseased apical periodontium and are classified as “successful” outcomes. Scores of 3.0, 4.0, and 5.0 signify the presence of apical radiolucency and are classified as “unsuccessful”. For multi-rooted teeth, the highest score among the roots was used as the final PAI score. Radiographic outcomes were categorized into three groups: Definite Success—Complete healing or significant reduction in peri-radicular radiolucency within two years. Probable Success—Stability or partial reduction in a radiolucency observed beyond one-year post-treatment. Failure—Persistent, new, or worsening radiolucency after one year, or clinical signs of infection such as swelling or sinus tract at recall.

## 3. Statistical Analysis

### Sample Size

For sample size calculation, we assumed a baseline endodontic treatment success rate (P_0_) of 0.9000 for the general population. This estimate is based on prior studies reporting success rates for nonsurgical root canal treatments ranging from 86% to 95% [8,9,13]. Given that our study is conducted in a supervised academic setting, we selected this conservative estimate to reflect the lower bound of the reported range while maintaining alignment with clinical expectations in similar environments. A sample size of 284 achieves 81% power to detect a difference of 0.0400 between the success rate of endodontic treatment performed by EIOH residents (P_1_) compared to the general population (P_0_), using a one-sided Z test that uses S(P_0_) to estimate the standard deviation. The target significance level is 0.0500, and the actual significance level achieved by this test is 0.0539.

Predictive models for patient-reported outcomes were fitted in one of two ways. For binary outcomes, Firth’s penalized logistic regressions were fitted. To estimate the odds ratio of experiencing an event (i.e., responding “yes”) as a function of covariates, Firth’s modification [16] to the standard logistic regression models improves the stability of estimands (e.g., reduces convergence rates and biases) that may otherwise be unstable due to rare events or perfect separation on some covariates. For ordinal (1–10 scale) outcomes, ordinal logistic regressions were fitted under a proportional odds assumption. These models assume that the coefficient for each covariate is the same between any pair of outcome responses. For both Firth’s logistic regression and the ordinal logistic regression models, these models were fitted in the R statistical programming environment (v4.4.2). Ninety-five percent confidence intervals are provided for each covariate and intercept term using profile likelihood intervals for Firth’s regressions and Wald intervals for the ordinal models. Corresponding significance tests are also reported using χ^2^ tests and z-tests, respectively.

## 4. Results

### 4.1. Characteristics of the Cohort

A total of 555 dental charts were reviewed and included in this study, all of which pertain to patients who received root canal therapy (RCT) in the AEGD department at the Eastman Institute for Oral Health. One chart (EXM05) included data regarding endodontic treatment performed at least one year prior to the recall exam. The study population ranged in age from 15 to 95 years, with a mean age of 49 years (median: 48; SD: 16.6). The majority were middle-aged, with the highest representation in the 35–39 age group (11.4%), followed by 40–44 (10.8%) and 45–49 (10.2%). Younger (15–24 years) and older (75+ years) age groups were underrepresented.

Most patients identified as White (46.13%) and African American (25.05%), with smaller representations from Asian (7.57%), Multiracial (0.90%), Native American (0.18%), and Native Hawaiian and Other Pacific Islander (0.18%) groups. Notably, 10.99% of participants declined to disclose their race, and 9.01% of the data was missing or non-applicable. Regarding ethnicity, 78.38% of participants identified as Not Hispanic, 11.89% as Hispanic, and 0.72% declined to report their ethnicity, while 9.01% of data was not reported.

The study population was predominantly female (63.78%), as shown in Table 1, which presents the demographic and medical conditions of patients showing that the majority do not report serious conditions. Most participants were free from chronic conditions such as diabetes (16.8%) and asthma (14.4%), (30.8%) reported alcoholism and (17.7%) smoking. Common issues included high blood pressure (26.1%) and arthritis (13.9%), while allergy-related conditions were more prevalent, such as penicillin allergy (13%) and hay fever (4.9%). Rare conditions, including HIV/AIDS (0.5%) and local anesthetic allergy (0.2%), were reported by a very small percentage of the population. Overall, the data suggests a population with a relatively low prevalence of serious health conditions but notable occurrences of chronic diseases and allergies.

### 4.2. Treatment Protocol Used in AEGD Clinic

All RCT procedures are performed by AEGD residents under faculty supervision, following a standardized protocol. Informed consent is obtained from each patient prior to treatment. After verifying the treatment site, profound local anesthesia is administered. Teeth are isolated with a rubber dam placement to prevent contamination and ensure a clean, safe, dry working field. Access cavity preparation is performed, and the working length is determined using a combination of electronic apex locators (Morita) and periapical radiographs. Root canal instrumentation is achieved using K files and Vortex rotary files (Dentsply Sirona, Charlotte, NC, USA) according to the manufacturer-recommended RPM and torque settings.

**Figure 1:**
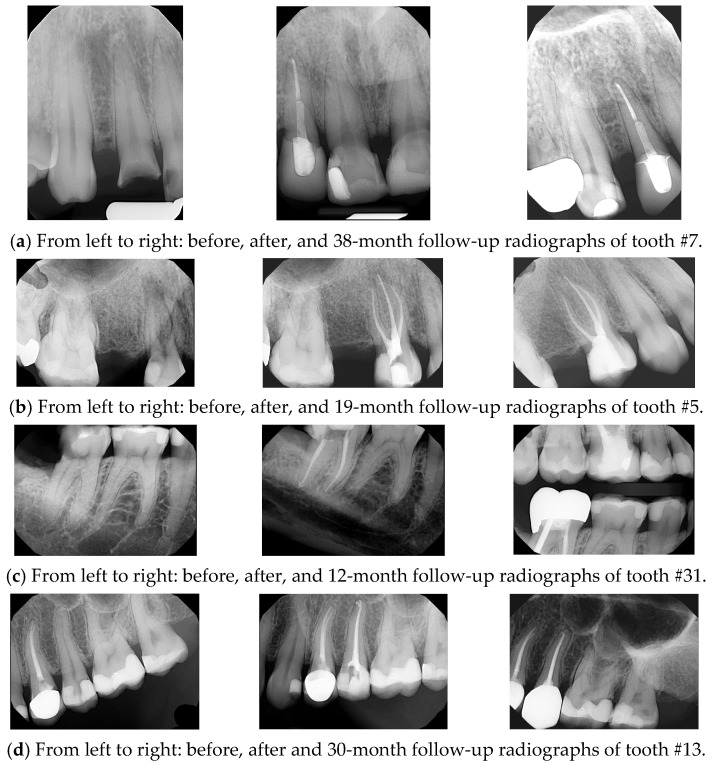

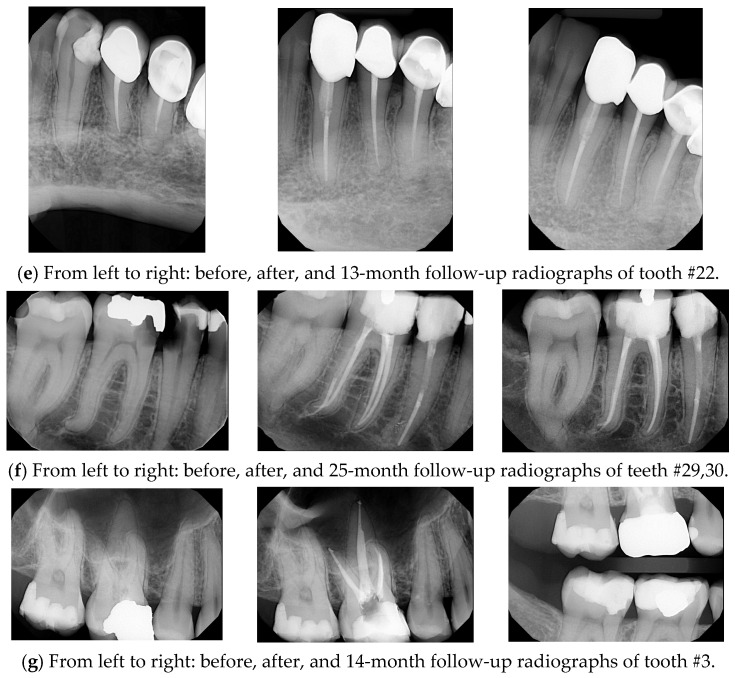
NSRCT cases performed by AEGD resident at EIOH.

Opious irrigation was applied in the canals throughout the procedure using 3% sodium hypochlorite (NaOCl) to ensure effective disinfection and debridement. Obturation is carried out using the warm vertical condensation technique, utilizing the Touch ’n Heat system (Kerr) and the Obtura backfill system (Spartan), in combination with a zinc oxide eugenol-based sealer (Kerr). A final periapical radiograph is taken to confirm the quality and length of the obturation. See Figure 1.

Our results showed that 100% of procedures were performed under rubber dam; a combination of hand filing and rotary techniques was employed in 91.7% of cases followed by hand filing alone (8.3%). The most used rotary system was the Tulsa system (98.8% of cases).

Obturation protocol: A total of 71.7% of cases involved backfilling. While vertical condensation was the most frequently used technique (86.3%), lateral condensation was used in 7.9% of cases. And a combination of vertical and lateral condensation was used in 5.7% of cases.

### 4.3. Treatment Outcomes and Success Rate

Among the 555 reports, a significant portion of responses to pain level after RCT were classified as “NA” (22.5%), suggesting either non-responses or cases where pain levels were not applicable or unreported. However, 430 (96%) patients reported no pain (Level 0). Mild to moderate pain (Levels 1–6) was infrequently reported, accounting for 3.3% of responses collectively. Severe pain (Levels 7–10) was exceedingly rare, with only one instance (0.2%) of maximum pain (Level 10). These findings indicate that most patients experienced minimal or no pain post-RCT, reflecting effective treatment and pain control in most cases.

Based on clinical and radiographic criteria, success was defined as the treated tooth being asymptomatic and functional with no significant periapical pathology observed during >12-month follow-up. Clinically, of the 555 records reviewed, 508 teeth (91.53%) exhibited no pain on percussion or palpation, no swelling, and no sinus tract, while no spontaneous pain was reported in 533 teeth (96.04%). Radiographically, periapical radiolucency was absent or reduced in 518 teeth (93.33%). Finally, the obturation quality was considered optimal (within 0–2 mm of the apex) in 88.11% of cases, and root resorption was absent in 98.38% of cases.

### 4.4. Complications of RCT

**Figure 2:**
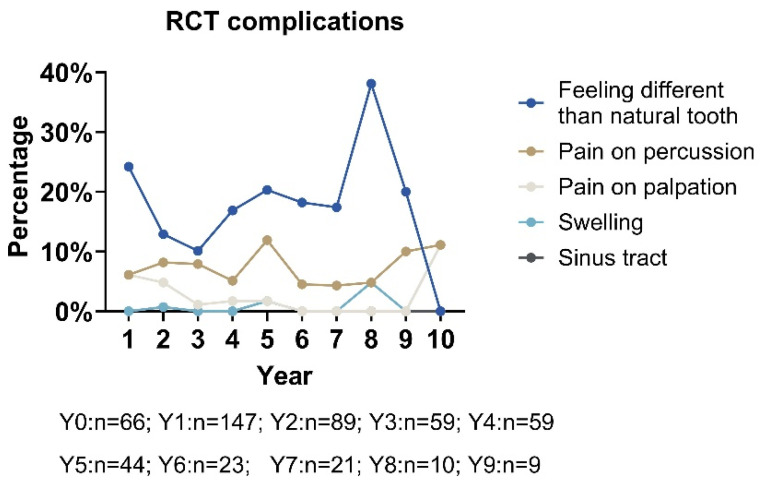
Complications of root canal teeth across 11 years (n = 529).

Over an 11-year period, the percentage of positive responses for various clinical outcomes like RCT tooth that feels different than natural teeth, pain on palpation, pain on percussion, the presence of a sinus tract, and the presence of swelling varied significantly (see Figure 2). The outcome “RCT tooth feels different than natural teeth” consistently exhibited the highest percentage of positive responses, peaking at 38.1% in Year 8 before declining sharply to 0% by Year 10. In contrast, “pain on palpation” demonstrated a gradual upward trend, reaching its highest prevalence of 11.1% in Years 10 and 11. Other outcomes, including “pain on percussion”, “presence of a sinus tract”, and “presence of swelling”, showed minimal or negligible positive responses, underscoring their limited clinical significance during the study period.

This line graph illustrates the percentage of positive responses for various outcomes over 11 years. These outcomes include that the RCT tooth feels different than natural teeth, pain on palpation, pain on percussion, the presence of a sinus tract, and the presence of swelling. The data highlights that different from natural tooth outcomes consistently have the highest percentage of positive responses, peaking at 38.1% in Year 8 before declining to 0% by Year 10. In contrast, the pain on palpation outcome exhibits a steady upward trend, reaching a peak of 11.1% in Years 10 and 11. Other outcomes, such as pain on percussion, the presence of a sinus tract, and swelling, show minimal or no significant responses, indicating limited relevance.

We have found strong statistical evidence (at the α = 0.05 level) suggesting that smoking/tobacco use, the presence of a pre-existing radiolucency, the periodontal health of the tooth, and whether the restoration is intact are all factors that influence whether a patient notices a difference between their natural teeth and a root canal treatment (RCT). The odds of a patient noticing a difference between their natural teeth and the RCT are about 2.4 times higher for those who smoke or use chewing tobacco compared to those who do not, assuming all other factors remain the same. The odds of noticing a difference are also about 2.4 times higher for those with a pre-existing radiolucency compared to those without, holding all other factors constant. For each additional level of severity in periodontal status (compared to “no periodontal issues”), the odds of noticing a difference increase by about 1.4 times, assuming other factors remain unchanged. The odds of noticing a difference are about 0.27 times lower for patients with an intact restoration compared to those with a defective one, while holding other factors constant.

In terms of pain frequency (as detailed in Table 2), a notable distinction is revealed between maxillary and mandibular teeth; mandibular molars demonstrated higher rates of “pain on percussion” (11.1%), while “pain on palpation” demonstrated more in maxillary molars (7.5%). Swelling was exceedingly rare across all regions, with the highest rates observed in mandibular anterior teeth (2.4%). Sinus tracts were also infrequent, with a solitary occurrence documented in mandibular molars (3.7%).

Considering the pain distribution among tooth locations (premolars, molars, and anterior teeth) and classifications (mandible and maxilla) in 430 participants, most of the participants reported no pain (level 0), with frequencies ranging from 91.4% to 100% across all groups, as outlined in Table 3. Pain levels greater than 0 were infrequent, occurring more commonly in molar and anterior teeth compared to premolars. Mild to moderate pain (levels 2–6) was observed in a small proportion of cases, particularly in maxillary premolars (5.3%) and mandibular molars (8.6%). Severe pain (level 10) was rare and reported exclusively in maxillary molars (3.13%).

### 4.5. Impact of Restoration Type and Integrity on Treatment Success and Patient

Satisfaction: The integrity of the final restoration was a significant predictor of treatment success. Teeth with intact restorations had 31.4 times higher odds of retention (*p* < 0.001), and patients reported significantly greater satisfaction with esthetics (OR = 21.92), function (OR = 52.37), and occlusion (OR = 52.37) when restorations were intact. Additionally, amalgam and composite restorations were associated with a 54% reduction in pain on percussion compared to crowns (OR = 0.46, *p* = 0.024). Although the timing of final restoration was recorded, it was not a significant predictor in the multivariate models and is discussed as a study limitation.

### 4.6. Predictive Factors Related to RCT Outcomes

#### Predictive Factors for RCT Outcomes When the Tooth Is Present

Our findings indicate a high success rate of RCT in terms of tooth retention (95.98%).

Several significant predictive factors influence the success of root canal treatment (RCT) when the tooth remains present, as detailed in Table 4. Among these factors, pre-existing periapical radiolucency emerged as a strong negative predictor of tooth retention. Specifically, the odds of the tooth being present are about 0.06 times lower compared to cases without pre-existing radiolucency.

This table presents the results of Firth’s logistic regression model evaluating factors associated with the presence of a tooth after root canal therapy (RCT): The analysis revealed that pre-existing radiolucency was associated with a 94% reduction in the odds of tooth retention compared to those without radiolucency. Intact restorations showed the highest positive impact, with a 31-fold increased odds of tooth survival. Conversely, the presence of cast or prefabricated metal posts was linked to a 99% reduced odds of tooth retention relative to having no post, while post in straight root were associated with a 70-fold increase in the odds of tooth survival relative to baseline. Factors such as age, gender, smoking, and periodontal status did not show statistically significant associations with tooth retention. Odds ratios less than 1 indicated a reduced odds of tooth retention, while those greater than 1 represented increased odds. These results underscore the importance of restoration integrity and post type in predicting long-term tooth retention after root canal therapy.

The condition of the final restoration was identified as a crucial determinant of treatment outcomes. Intact restorations significantly improved the probability of tooth retention. The odds of the tooth being present are about 31.4 times higher when the final restoration is intact compared to when it is defective.

The type of post used during treatment also played a significant role in tooth retention after RCT. The use of metal posts, including cast and prefabricated metal designs, was associated with a 0.0136-fold decrease in the odds of tooth retention compared to cases without post placement. Conversely, fiber posts did not exhibit a statistically significant impact on survival rates. Additionally, the anatomical positioning of the post was an influential factor, with posts placed in straight roots conferring a 69.55 times greater chance of tooth survival compared to those positioned in curved roots.

### 4.7. Predictive Factors for Pain on Percussion

While a small group of patients (8.67%) experienced pain on percussion, key factors influencing the odds of pain on percussion following RCT are indicated in Table 5. The periodontal status of the treated tooth was a significant predictor, demonstrating a positive association with pain on percussion. Each progressive decline in periodontal health (ranging from no periodontal disease to gingivitis, mild periodontitis, moderate periodontitis, and severe periodontitis) was associated with a 1.74 times increase in the odds of experiencing pain on percussion. Additionally, the type of final restoration placed after RCT was a significant predictor of post-treatment pain on percussion. The use of an amalgam or composite restoration was associated with a 0.46-fold reduction in the odds of experiencing pain on percussion compared to cases restored with a crown.

Periodontal status of the tooth was significantly associated with pain on percussion (*p* = 0.00230), indicating that worse periodontal health increases the odds of experiencing pain. The type of restoration showed statistical significance (*p* = 0.02411), suggesting that defective restorations may contribute to increased pain on percussion. Other covariates, such as age, gender, race, number of appointments, post type, and radiographic evaluation, were not statistically significant.

### 4.8. Predictive Factors for Patient Satisfaction with Esthetics

The integrity of the final restoration was identified as the most critical predictor of patient satisfaction with esthetics (Table 6). Patients were significantly more likely to report higher esthetic satisfaction when the restoration remained intact, with 21.92-fold increase in positive satisfaction compared to cases with defective restorations.

Restoration integrity was a significant predictor (*p* < 0.001) for improved clinical outcomes, with teeth having intact restorations showing a strong positive association. Intercepts at satisfaction levels ≤7 vs. ≥8, ≤8 vs. ≥9, and ≤9 vs. ≥10 were statistically significant (*p* < 0.01 and *p* < 0.001), highlighting their association with higher satisfaction levels. Other covariates such as age, race, location, smoking, and radiographic evaluation showed no significant associations with clinical outcomes.

### 4.9. Predictive Factors for Patient Satisfaction with Function

As outlined in Table 7, several key factors influence patient-reported satisfaction with the function of the RCT-treated tooth. Patients without medical conditions had 3.85 times greater odds of reporting higher functional satisfaction compared to those with medical conditions. Pre-existing radiolucency and periodontal issues were negatively associated with satisfaction, with the odds of satisfaction being 0.46 times lower for those with a pre-existing radiolucency and 0.41 times lower as periodontal conditions worsened.

Pre-existing radiolucency was significantly associated with reduced odds of clinical success (*p* = 0.00604), suggesting a negative impact on outcomes. Periodontal status was highly significant (*p* < 0.001), indicating that worse periodontal health decreases the odds of positive treatment outcomes. An intact restoration showed a very strong positive association with treatment success (*p* < 0.001), emphasizing its importance for long-term clinical stability. A post in straight root was also significantly associated with improved clinical outcomes (*p* = 0.00741), whereas other post types and restoration categories were not statistically significant. Intercepts at satisfaction levels ≤5 vs. ≥6, ≤6 vs. ≥7, ≤7 vs. ≥8, ≤8 vs. ≥9, and ≤9 vs. ≥10 showed statistical significance (*p* < 0.05 to *p* < 0.001), indicating a stepwise increase in positive treatment satisfaction with higher levels of restoration quality or improved radiographic outcomes.

Restoration integrity was found to be the strongest predictor of functional satisfaction. The odds of reporting higher satisfaction were 52.37 times greater for patients with intact restorations compared to those with defective restorations. Additionally, the odds of higher satisfaction with tooth function were 2.86 times greater when a post was used in a straight root, suggesting a positive correlation between proper post placement and functional satisfaction.

### 4.10. Predictive Factors for Patient Satisfaction with Occlusion

Predictive factors for patient satisfaction with occlusion following RCT (Table 8) include overall health status, with patients without medical conditions having 3.85 times greater odds of being satisfied with occlusion compared to those with medical conditions.

Pre-existing radiolucency was significantly associated with reduced odds of positive treatment outcomes (*p* = 0.00604), indicating a negative impact on root canal success. Periodontal status was highly significant (*p* < 0.001), demonstrating that worse periodontal health is strongly linked to poorer treatment outcomes. An intact restoration showed the most substantial positive association with successful treatment (*p* < 0.001). A post in straight root was significantly associated with better outcomes (*p* = 0.01788), as well as the type of restoration (*p* = 0.04), while post types did not show statistical significance. Intercepts at satisfaction levels ≤5 vs. ≥6, ≤6 vs. ≥7, ≤7 vs. ≥8, ≤8 vs. ≥9, and ≤9 vs. ≥10 were statistically significant (*p* < 0.01 to *p* < 0.001), indicating stepwise improvements in outcomes with increasing treatment quality and post stability.

Pre-existing radiolucency and poor periodontal health negatively impacted occlusal satisfaction, with the odds of satisfaction being 0.463 times lower for those with pre-existing radiolucency and 0.406 times lower for each additional level of periodontal severity (relative to no periodontal disease).

Patients reported significantly higher satisfaction when the final restoration was a crown, while restoration integrity and the presence of a post in a straight canal also positively influenced perceived occlusal outcomes. Patients with an intact restoration had 52.37 times greater odds of reporting higher satisfaction with occlusion, while the presence of a post in a straight root was associated with 2.869 times greater odds of higher satisfaction, holding all else constant.

## 5. Discussions

### 5.1. Comparisons of RCT Success Rate to Published Studies

The findings of our study highlight the high success rate of NSRCTs performed by AEGD residents, aligning with previously published success rates ranging from 86% to 95% [13]. Endodontic treatment success rates are influenced by several factors, including the criteria used to define success, the duration of follow-up, and the techniques employed. In the literature, success is frequently defined by the absence of clinical signs and symptoms (e.g., pain, swelling, or sinus tract) and the radiographic evidence of healing or lack of progression in periapical radiolucency, with reported success rates for nonsurgical root canal treatments ranging from 86% to 95%, based on proper diagnosis, adherence to treatment protocols, and patient-specific variables [13]. Over time, success rates decline, underscoring the importance of extended follow-up periods for accurate assessment, with success rates reported to range from 85% to 95% at 2–4 years, decreasing to 80% to 90% at 4–6 years [8]. The success rate observed in our study reinforces the effectiveness of the structured AEGD residency training program at EIOH in ensuring quality endodontic care.

Our results highlight the importance of defining and reinforcing core competencies in endodontic education to improve clinical outcomes among general dentists. Recent research emphasizes the need for consistent standards and faculty-driven benchmarks in residency training programs [17]. This study also collected information on the type of instrumentation used (manual, rotary, or both), which was included among the assessed variables. However, the specific impact of instrumentation technique, treatment duration, and materials used on success outcomes was not found to be statistically significant in the preliminary multivariate modeling. These variables may still influence treatment outcomes, and their effects warrant further investigation. We acknowledge that treatment duration and specific material types used during procedures were not included in our statistical analysis, representing a limitation of this study. Future research should explore these parameters more closely to determine their potential contribution to treatment success.

### 5.2. Comparisons of RCT Complications to Published Studies

This study’s findings support and build on previous research about root canal therapy (RCT) outcomes, providing new insights into both clinical results and patient feedback. The observed association between intact restorations and increased chance of tooth retention supports previous research emphasizing the critical role of coronal restoration integrity in post-RCT survival [18,19]. Similarly, the adverse effects of cast or prefabricated posts on tooth retention are consistent with the findings of Mentink and colleagues [20], who reported an increased risk of tooth fracture associated with these types of posts.

The observed relationship between older age, improved periodontal health, and reduced post-endodontic treatment pain supports the findings of Ng’s study [13], which identified age as a predictor of less post-treatment discomfort. The role of periodontal conditions as a factor in RCT success has also been documented [21]. Furthermore, while limited studies have directly examined the association between post type and pain, Mannocci highlighted the biomechanical benefits of straight fiber posts in minimizing stress, findings that align with this study’s observation of reduced pain in cases utilizing straight posts [22].

An important consideration in the assessment of root canal treatment (RCT) post-op complications is the persistent prevalence of apical periodontitis (AP) among endodontically treated teeth. A systematic review and meta-analysis by Jakovljevic et al. (2020) analyzed cross-sectional studies published between 2012 and 2020 and reported a high prevalence of AP in root-filled teeth, with rates varying from 31.5% to 57.8%, depending on location and radiographic assessment methods used [23]. This finding suggests that, while root canal therapy is often deemed successful based on clinical criteria or tooth retention, radiographically silent or unresolved AP may still be present in a substantial proportion of cases. Compared to their findings, our study reports a significantly lower radiographic prevalence of persistent periapical pathology (6.67%), indicating favorable outcomes within our academic AEGD program. This contrast could reflect differences in operator training, restorative integrity, follow-up duration, or diagnostic criteria. Nonetheless, it underscores the critical need for rigorous radiographic evaluation and long-term follow-up to more accurately assess treatment outcomes and to ensure that clinically asymptomatic teeth are truly free of underlying periapical inflammation.

Overall, pain prevalence in our study varied between maxillary and mandibular molars, as well as between premolars, in terms of pain on percussion. Additionally, differences were observed between maxillary and mandibular premolars regarding pain on palpation. However, our findings are consistent with the study by Sadaf et al. [24], which reported lower pain frequency in maxillary molars compared to mandibular molars. Mandibular molars are more prone to postoperative pain due to denser bone and more complex canal anatomy, which hinders pressure relief. In contrast, the less dense bone around maxillary molars may help reduce pain after treatment.

Our findings indicate that mild to moderate pain (levels 2–6) was most reported in maxillary premolars (5.3%) and mandibular molars (8.6%). These results align with the study by Klasser et al., which found that persistent neuropathic pain occurred in 7% of patients following single-tooth nonsurgical root canal treatment. Their study also reported a higher prevalence in middle-aged individuals (mean age 50.6), with no sex predilection, and a greater occurrence in the mandibular arch [25].

The patient perception of tooth differences was influenced by factors such as smoking and pre-existing radiolucency, which have been shown to impair oral healing and affect patient outcomes [26]. Intact restorations reduced this likelihood, reinforcing the importance of restoration quality. These results also align with Torabinejad’s study [27], highlighting the role of functional and esthetic success in patient perceptions of RCT-treated teeth.

Periodontal health and restoration integrity played significant roles in reducing pain on percussion and palpation, echoing the findings of Ng et al. [13] and Saunders and Saunders [28], who emphasized the importance of these factors in minimizing post-treatment complications. Patient-reported outcomes, including satisfaction with esthetics, function, and occlusion, were consistently higher when restorations were intact. This is also in agreement with the observations of Sequeira-Byron et al. [29], who highlighted the relationship between restoration quality and overall patient satisfaction. The role of straight posts in improving functional satisfaction reflects findings by Morgano [30] regarding the impact of post-placement on occlusal dynamics.

### 5.3. The Impact of Pre-Existing Conditions, Periodontal Health, and Restoration Integrity on Patient Satisfaction with RCT Outcomes

One of the most consistent findings across the analyses is the significant influence of pre-existing conditions and periodontal health on patient satisfaction. Patients with pre-existing conditions, including radiolucency or poor periodontal health, were more likely to report lower satisfaction across all outcomes—function, occlusion, and esthetics. These results suggest that pre-existing conditions not only impact clinical success but also shape how patients perceive the effectiveness of their treatment. This underscores the importance of comprehensive pre-treatment evaluations to identify and manage such conditions before initiating RCT. The integrity of the restoration emerged as the most significant predictor of positive patient-reported outcomes. An intact restoration was strongly associated with higher satisfaction across all dimensions of RCT success.

Our results are consistent with the study by Gillen et al. [31], which demonstrated that the odds of apical periodontitis healing significantly improve when both root canal and restorative treatments are adequately performed. This finding highlights the importance of meticulous restorative care and follow-up to ensure long-term durability and functionality of the treated tooth. Furthermore, procedural factors such as the use of post in straight root were found to positively influence functional and occlusal satisfaction, suggesting that technical decisions for the restoration of the RCT-treated teeth play a crucial role in shaping patient-reported outcomes. Additionally, satisfaction with esthetics was also influenced by the intact condition of the tooth, aligning with findings related to functional and occlusal satisfaction.

However, other demographic and procedural factors, such as patient age, gender, or tooth classification, were not consistently significant predictors of satisfaction, suggesting that structural and clinical integrity may outweigh demographic considerations in determining patient perceptions. These findings emphasize the need for a holistic approach to RCT that prioritizes both clinical success and patient satisfaction. By addressing pre-existing conditions, maintaining periodontal health, and ensuring the integrity of restorations, clinicians can optimize both functional and esthetic outcomes. Moreover, the results highlight the value of patient-centered care and tailored treatment planning in enhancing the overall experience of RCT. Future studies should further explore these relationships, particularly the impact of specific restorative materials and techniques, to refine best practices in endodontic care.

## 6. Limitations

While this study draws significant parallels, certain limitations were noted. Several covariates (e.g., cleaning and shaping, obturation technique, rotary system) were omitted from the model due to high levels of imbalance causing numerical and statistical instabilities.

Rare outcomes, such as swelling and sinus tract formation, had limited statistical power due to their low prevalence, even though previous research has underscored their importance as indicators of treatment success [32]. Furthermore, the application of Firth’s penalized logistic regression enabled the sophisticated modeling of sparse data, but this methodology has not been widely employed in earlier studies.

The findings are most reflective of middle-aged individuals and may have limited applicability to other age groups. It is also noteworthy that all 555 records analyzed were from patients who attended recall evaluations at least the one-year post-treatment, thereby representing compliant patients, which may limit the generalizability of the findings.

While the timing of final restoration placement was documented and considered during initial variable selection, it did not emerge as a significant predictor of clinical success or patient satisfaction in our regression models. This may be due to confounding or narrow variation in timing across cases. Future studies with a broader temporal distribution may further clarify the impact of restoration timing on long-term outcomes.

## 7. Conclusions

This study indicates that post-doctoral general dentistry residents under supervision and with structured training can perform RCT with high success. It also confirms the critical importance of restoration quality, periodontal health, and post type in RCT success, aligning well with the established literature. Future studies could address gaps in rare outcome measures and further explore patient-reported outcomes to build a more comprehensive framework for RCT evaluation.

Key predictors of clinical success, including restoration integrity, periodontal health, and post placement, were found to significantly influence outcomes such as tooth retention and pain. These factors are largely modifiable and should inform clinical decision making and resident training curricula.

This study confirms that post-doctoral general dentistry residents, under structured supervision and training, achieved a clinical success rate of 91.5% and a radiographic success rate of 93.3%, supporting this study’s hypothesis that AEGD residents can achieve success rates equal to or exceeding 92%. The findings reinforce the importance of restoration integrity, periodontal health, and appropriate post selection in achieving optimal RCT outcomes. These results underscore the value of comprehensive clinical training in postgraduate dental education and suggest directions for enhancing endodontic education and clinical protocols.

## Figures and Tables

**Table 1:** Demographics of patients included in this study (*n* = 555).

Characteristic	Number (%)
**Gender**	
Female	354 (63.78%)
Male	201 (36.22%)
**Race**	
White	256 (46.13%)
African American	139 (25.05%)
Unknown	111 (20%)
Asian	42 (7.57%)
Multiracial	5 (0.90%)
Native American	1 (0.18%)
Native Hawaiian and Other Pacific Islander	1 (0.18%)
**Ethnicity**	
Not Hispanic	435 (78.38%)
Hispanic	66 (11.89%)
Unknown	54 (9.73%)
**Medical Condition**	
Alcoholism	171 (30.8%)
High blood pressure	145 (26.1%)
Diabetes	93 (16.8%)
Asthma	80 (14.4%)
Arthritis	77 (13.9%)
Psychiatric care	77 (13.9%)
Anemia	34 (6.1%)
Has/had cancer or tumor	23 (4.1%)
Artificial joint or valve	17 (3.1%)
Heart murmur / Heart valve	19 (3.4%)
Kidney disease	15 (2.7%)
Angina or heart disease	14 (2.5%)
Hepatitis	11 (2.0%)
Epilepsy	9 (1.6%)
Herpes	7 (1.3%)
TB	4 (0.7%)
Pacemaker	3 (0.5%)
AIDS/HIV	3 (0.5%)
**Allergies**	
No known allergy	356 (64.1%)
Penicillin allergy	72 (13.0%)
Allergies or hives	58 (10.5%)
Codeine allergy	31 (5.6%)
Latex allergy	31 (5.6%)
Sulfa allergy	29 (5.2%)
Hay fever or sinus trouble	27 (4.9%)
Aspirin allergy	15 (2.7%)
Barbiturate or sedative allergy	3 (0.5%)
Local anesthetic allergy	1 (0.2%)
**Smoking**	
Smoke or use chewing tobacco	98 (17.7%)

**Table 2:** Complications of root canal teeth by locations (n = 531).

Complications	Premolar (Mandible) n = 77	Premolar (Maxilla) n = 142	Molar (Mandible) n = 54	Molar (Maxilla) n = 40	Anterior (Mandible) n = 41	Anterior (Maxilla) n = 177
Pain on Percussion	2 (2.6%)	13 (9.2%)	6 (11.1%)	3 (7.5%)	3 (7.3%)	13 (7.3%)
Pain on Palpation	0 (0.0%)	4 (2.8%)	3 (5.6%)	3 (7.5%)	1 (2.4%)	5 (2.8%)
Swelling	0 (0.0%)	0 (0.0%)	1 (1.9%)	0 (0.0%)	1 (2.4%)	1 (0.6%)
Sinus Tract	0 (0.0%)	0 (0.0%)	2 (3.7%)	0 (0.0%)	0 (0.0%)	0 (0.0%)

**Table 3:** Level of pain by location and classification (with percentages) (n = 430).

Location	Classification	Level 0	Level 1	Level 2	Level 3	Level 4	Level 5	Level 6	Level 7	Level 8	Level 9	Level 10
Premolar	Mandible (67)	100%	0%	0%	0%	0%	0%	0%	0%	0%	0%	0%
	Maxilla (113)	94.7%	0%	0%	2.65%	0.88%	1.77%	0%	0%	0%	0%	0%
Molar	Mandible (35)	91.4%	0%	0%	2.86%	2.86%	2.86%	0%	0%	0%	0%	0%
	Maxilla (32)	93.75%	0%	0%	0%	3.13%	0%	0%	0%	0%	0%	3.13%
Anterior	Mandible (37)	91.89%	0%	2.7%	0%	0%	2.7%	2.7%	0%	0%	0%	0%
	Maxilla (146)	97.95%	0%	0%	0.68%	0.68%	0%	0.68%	0%	0%	0%	0%

**Table 4:** Logistic regression for root canal outcome: tooth present (Y/N) (n = 323).

Covariate	Est.	Std. Error	95% PL CI	$\chi^{2}\left(1\right)$	p-Value	Sig.
(Intercept)	1.9608	2.0937	(−4.1336, 8.8225)	0.4853	0.48600	
Age	−0.0034	0.0214	(−0.0599, 0.0521)	0.0162	0.89870	
Male (Y/N)	0.6294	0.6906	(−1.0586, 3.4043)	0.5089	0.47560	
Non-white (Y/N)	0.8360	0.7384	(−1.006, 3.1707)	0.7962	0.37220	
Hispanic (Y/N)	−1.0509	0.9592	(−4.2243, 1.7484)	0.6281	0.42800	
No conditions (Y/N)	0.3567	0.8379	(−2.3237, 2.8713)	0.1057	0.74510	
Smoke/use chewing tobacco (Y/N)	−1.5247	0.8164	(−5.8464, 0.6966)	1.8092	0.17860	
Location is maxilla (Y/N)	−0.2932	0.7556	(−2.3902, 1.6981)	0.0893	0.76500	
Classification is molar (Y/N)	0.3022	0.9378	(−1.9945, 2.8381)	0.0695	0.79210	
Classification is anterior (Y/N)	−0.1265	0.6705	(−1.7774, 1.4741)	0.0252	0.87380	
Num. of appts. to complete RCT	0.0516	0.2397	(−0.5757, 0.9136)	0.0279	0.86720	
Pre-existing radiolucency (Y/N)	−2.7398	1.0058	(−8.3087, −0.3886)	5.5972	0.01799	*
Periodontal status of the tooth	−0.3360	0.3331	(−1.4609, 0.547)	0.5584	0.45490	
Type of restoration	0.3086	0.4836	(−0.8302, 1.507)	0.2978	0.58530	
Restoration is intact (Y/N)	3.4463	0.8232	(1.5963, 7.895)	17.6380	0.00003	***
Metal post (Y/N)	−4.2963	1.5272	(−13.3039, −0.7049)	6.3619	0.01166	*
Fiber post (Y/N)	−1.4415	1.5255	(−7.172, 4.2949)	0.3640	0.54630	
Post in straight root (Y/N)	4.2421	1.5293	(0.4856, 13.8117)	5.3571	0.02064	*
Post in curved root (Y/N)	2.0990	1.4224	(−1.648, 8.005)	1.1253	0.28880	
Radiographic eval.	−0.0153	0.3327	(−0.8575, 1.0244)	0.0012	0.97240	

* < 0.05, *** < 0.001.

**Table 5:** Logistic regression for root canal outcome: pain on percussion (n = 323).

Covariate	Est.	Std. Error	95% PL CI	$\chi^{2}\left(1\right)$	p-Value	Sig.
(Intercept)	−0.2105	1.2035	(−2.7956, 2.3414)	0.0261	0.87150	
Age	−0.0104	0.0130	(−0.0386, 0.0173)	0.5480	0.45920	
Male (Y/N)	0.1046	0.4309	(−0.8418, 1.0126)	0.0496	0.82380	
Non-white (Y/N)	−0.5281	0.4188	(−1.4473, 0.35)	1.3789	0.24030	
Hispanic (Y/N)	−0.7689	0.7863	(−3.0065, 0.7129)	0.8954	0.34400	
No conditions (Y/N)	−0.4554	0.5018	(−1.6218, 0.559)	0.7316	0.39240	
Smoke/use chewing tobacco (Y/N)	0.4330	0.4487	(−0.548, 1.3528)	0.7880	0.37470	
Location is maxilla (Y/N)	0.0061	0.4480	(−0.9383, 0.9901)	0.0002	0.99000	
Classification is molar (Y/N)	−0.3027	0.5678	(−1.5787, 0.8711)	0.2458	0.62010	
Classification is anterior (Y/N)	−0.2056	0.4546	(−1.1837, 0.7596)	0.1754	0.67540	
Num. of appts. to complete RCT	0.2063	0.1508	(−0.1388, 0.518)	1.4651	0.22610	
Pre-existing radiolucency (Y/N)	0.1172	0.3926	(−0.7277, 0.9643)	0.0752	0.78390	
Periodontal status of tooth	0.5550	0.1684	(0.2045, 0.9057)	9.2942	0.00230	**
Type of restoration	−0.7659	0.3139	(−1.4267, −0.1024)	5.0869	0.02411	*
Restoration is intact (Y/N)	−0.4171	0.4534	(−1.3524, 0.5843)	0.7037	0.40150	
Metal post (Y/N)	0.5391	0.6372	(−0.8875, 1.8587)	0.5954	0.44030	
Fiber post (Y/N)	0.4256	0.5658	(−0.8079, 1.6544)	0.4711	0.49250	
Post in straight root (Y/N)	−0.8536	0.5024	(−1.9465, 0.1965)	2.5382	0.11110	
Post in curved root (Y/N)	−0.6488	0.7831	(−2.5202, 0.9103)	0.6194	0.43130	
Radiographic Eval.	−0.2347	0.2133	(−0.7425, 0.1974)	1.0660	0.30190	

* < 0.05, ** < 0.01

**Table 6:** Ordinal logistic regression for patient-reported outcome: satisfaction with esthetics (n = 321).

Covariate	Est.	Std. Error	95% CI	z Stat.	p-Value	Sig.
Age	0.0057	0.0080	(0.0214, −0.01)	0.7172497	0.47320	
Male (Y/N)	0.0714	0.2477	(0.5569, −0.4141)	0.2882467	0.77320	
Non-white (Y/N)	0.3396	0.2260	(0.7826, −0.1034)	1.5028592	0.13290	
Hispanic (Y/N)	−0.0395	0.3015	(0.5514, −0.6304)	−0.1309459	0.89580	
Asthma (Y/N)	−0.2985	0.3171	(0.323, −0.92)	−0.9414325	0.34650	
No conditions (Y/N)	−0.0996	0.2755	(0.4404, −0.6396)	−0.3614109	0.71780	
Smoke/use chewing tobacco (Y/N)	0.0235	0.2909	(0.5937, −0.5467)	0.0808452	0.93560	
Location is maxilla (Y/N)	−0.0041	0.2420	(0.4702, −0.4784)	−0.0168121	0.98660	
Classification is molar (Y/N)	−0.1355	0.3123	(0.4766, −0.7476)	−0.4336780	0.66450	
Classification is anterior (Y/N)	−0.2297	0.2389	(0.2385, −0.6979)	−0.9614684	0.33630	
Restoration is intact (Y/N)	3.0872	0.3357	(3.7452, 2.4292)	9.1957321	0.00000	***
Radiographic Eval.	0.0426	0.1075	(0.2533, −0.1681)	0.3966283	0.69160	
Intercept	Est.	Std. Error	95% CI	z stat.	p-value	Sig.
01	−0.9333	0.6156	(0.2733, −2.1399)	−1.5161329	0.12950	
12	−0.7680	0.6108	(0.4292, −1.9652)	−1.2574571	0.20860	
23	−0.6914	0.6087	(0.5017, −1.8845)	−1.1358350	0.25600	
34	−0.5507	0.6055	(0.6361, −1.7375)	−0.9094519	0.36310	
45	−0.0809	0.6002	(1.0955, −1.2573)	−0.1347940	0.89280	
56	0.5793	0.6021	(1.7594, −0.6008)	0.9620442	0.33600	
67	1.1759	0.6085	(2.3686, −0.0168)	1.9322787	0.05333	
78	1.8469	0.6180	(3.0582, 0.6356)	2.9887187	0.00280	**
89	2.6237	0.6264	(3.8514, 1.396)	4.1882417	0.00003	***
910	3.1192	0.6292	(4.3524, 1.886)	4.9573202	0.00000	***

** < 0.01, *** < 0.001.

**Table 7:** Ordinal logistic regression for patient-reported outcome: satisfaction with function (n = 255).

Covariate	Est.	Std. Error	95% CI	z Stat	p-Value	Sig.
Age	0.0127	0.0098	(−0.0065, 0.0319)	1.2989196	0.19400	
Male (Y/N)	0.3717	0.3360	(−0.2869, 1.0303)	1.1062783	0.26860	
Non-white (Y/N)	0.4427	0.2955	(−0.1365, 1.0219)	1.4982001	0.13410	
Hispanic (Y/N)	−0.7456	0.3879	(−1.5059, 0.0147)	−1.9219718	0.05461	
Asthma (Y/N)	−0.0133	0.3883	(−0.7744, 0.7478)	−0.0343227	0.97260	
No conditions (Y/N)	1.3490	0.4201	(0.5256, 2.1724)	3.2114060	0.00132	**
Smoke/use chewing tobacco (Y/N)	0.3131	0.3668	(−0.4058, 1.032)	0.8536207	0.39330	
Location is maxilla (Y/N)	−0.0376	0.3037	(−0.6329, 0.5577)	−0.1238733	0.90140	
Classification is molar (Y/N)	−0.4837	0.3995	(−1.2667, 0.2993)	−1.2107436	0.22600	
Classification is anterior (Y/N)	−0.1875	0.3167	(−0.8082, 0.4332)	−0.5922568	0.55370	
Num. of appts. to complete RCT	0.0381	0.1237	(−0.2044, 0.2806)	0.3082347	0.75790	
Pre-existing radiolucency (Y/N)	−0.7699	0.2804	(−1.3195, −0.2203)	−2.7454762	0.00604	**
Periodontal status of tooth	−0.9005	0.1498	(−1.1941, −0.6069)	−6.0136463	0.00000	***
Type of restoration	0.4214	0.2497	(−0.068, 0.9108)	1.6875502	0.09150	
Restoration is intact (Y/N)	3.9584	0.4488	(3.0788, 4.838)	8.8207555	0.00000	***
Metal post (Y/N)	−0.3082	0.4658	(−1.2212, 0.6048)	−0.6617978	0.50810	
Fiber post (Y/N)	−0.2891	0.3652	(−1.0049, 0.4267)	−0.7916293	0.42860	
Post in straight root (Y/N)	1.0538	0.3936	(0.2823, 1.8253)	2.6776059	0.00741	**
Post in curved root (Y/N)	0.8671	0.5773	(−0.2644, 1.9986)	1.5021694	0.13310	
Radiographic eval	0.2405	0.1437	(−0.0412, 0.5222)	1.6740322	0.09412	
Intercept	Est.	Std. Error	95% CI	z stat.	p-value	Sig.
01	0.8366	0.9597	(−1.0444, 2.7176)	0.8716637	0.38340	
12	1.0178	0.9571	(−0.8581, 2.8937)	1.0633914	0.28760	
23	1.1031	0.9562	(−0.7711, 2.9773)	1.1535688	0.24870	
34	1.1031	0.9562	(−0.7711, 2.9773)	1.1535691	0.24870	
45	1.2674	0.9553	(−0.605, 3.1398)	1.3267701	0.18460	
56	2.0704	0.9612	(0.1864, 3.9544)	2.1541018	0.03123	*
67	2.7968	0.9730	(0.8897, 4.7039)	2.8743878	0.00405	**
78	3.5436	0.9838	(1.6154, 5.4718)	3.6021003	0.00032	***
89	4.4770	0.9978	(2.5213, 6.4327)	4.4867608	0.00001	***
910	5.2657	1.0118	(3.2826, 7.2488)	5.2041119	0.00000	***

* < 0.05, ** < 0.01, *** < 0.001.

**Table 8:** Ordinal logistic regression for patient-reported outcome: satisfaction with occlusion (n = 251).

Covariate	Est.	Std. Error	95% CI	z Stat	p-Value	Sig.
Age	0.0181	0.0099	(−0.0013, 0.0375)	1.8237583	0.06954	
Male (Y/N)	0.1276	0.3325	(−0.5241, 0.7793)	0.3836267	0.70160	
Non-white (Y/N)	0.4669	0.3002	(−0.1215, 1.0553)	1.5549825	0.12140	
Hispanic (Y/N)	−0.6464	0.3943	(−1.4192, 0.1264)	−1.6392154	0.10260	
Asthma (Y/N)	−0.0011	0.3885	(−0.7626, 0.7604)	−0.0028486	0.99770	
No conditions (Y/N)	1.4308	0.4240	(0.5998, 2.2618)	3.3743472	0.00087	***
Smoke/use chewing tobacco (Y/N)	0.2242	0.3675	(−0.4961, 0.9445)	0.6101076	0.54240	
Location is maxilla (Y/N)	0.0758	0.3060	(−0.524, 0.6756)	0.2476545	0.80460	
Classification is molar (Y/N)	−0.2434	0.3993	(−1.026, 0.5392)	−0.6095559	0.54280	
Classification is anterior (Y/N)	−0.1647	0.3199	(−0.7917, 0.4623)	−0.5148864	0.60710	
Num. of appts. to complete RCT	0.0222	0.1270	(−0.2267, 0.2711)	0.1749472	0.86130	
Pre-existing radiolucency (Y/N)	−0.7448	0.2835	(−1.3005, −0.1891)	−2.6273969	0.00921	**
Periodontal status of tooth	−0.9138	0.1462	(−1.2004, −0.6272)	−6.2482193	0.00000	***
Type of restoration	0.5177	0.2520	(0.0238, 1.0116)	2.0545722	0.04110	*
Restoration is intact (Y/N)	3.5411	0.4311	(2.6961, 4.3861)	8.2132800	0.00000	***
Metal post (Y/N)	−0.1156	0.4762	(−1.049, 0.8178)	−0.2428246	0.80840	
Fiber post (Y/N)	−0.1695	0.3725	(−0.8996, 0.5606)	−0.4550398	0.64950	
Post in straight canal (Y/N)	0.9451	0.3961	(0.1687, 1.7215)	2.3859882	0.01788	*
Post in curved canal (Y/N)	0.5328	0.5786	(−0.6013, 1.6669)	0.9207637	0.35820	
Radiographic eval	0.0909	0.1420	(−0.1874, 0.3692)	0.6403563	0.52260	
Intercept	Est.	Std. Error	95% CI	z stat	p-value	Sig.
01	0.5360	0.9799	(−1.3845, 2.4566)	0.5471	0.5843	
12	0.7573	0.9749	(−1.1536, 2.6682)	0.7768	0.4373	
23	0.8564	0.9732	(−1.0511, 2.7638)	0.8800	0.3789	
34	0.9476	0.9715	(−0.9566, 2.8519)	0.9754	0.3294	
45	1.1171	0.9691	(−0.7824, 3.0165)	1.1527	0.2490	
56	2.1192	0.9676	(0.2227, 4.0157)	2.1902	0.0285	*
67	2.7850	0.9762	(0.8716, 4.6984)	2.8529	0.0043	***
78	3.5558	0.9886	(1.6182, 5.4934)	3.5969	0.0003	***
89	4.2135	0.9980	(2.2574, 6.1696)	4.2219	0.0000	***
910	5.0653	1.0117	(3.0823, 7.0484)	5.0065	0.0000	***

* < 0.05, ** < 0.01, *** < 0.001.

## Data Availability

The data used in this study are available upon reasonable request from the corresponding author.
